# An Electromagnetically Excited Silicon Nitride Beam Resonant Accelerometer

**DOI:** 10.3390/s90301330

**Published:** 2009-02-26

**Authors:** Deyong Chen, Zhengwei Wu, Lei Liu, Xiaojing Shi, Junbo Wang

**Affiliations:** Institute of Electronics, Chinese Academy of Sciences, State Key Laboratories of Transducer Technology, Beijing, P.R. China

**Keywords:** Resonant accelerometer, Modeling, Silicon rich nitride, Triple beam, Electromagnetic excitation

## Abstract

A resonant microbeam accelerometer of a novel highly symmetric structure based on MEMS bulk-silicon technology is proposed and some numerical modeling results for this scheme are presented. The accelerometer consists of two proof masses, four supporting hinges, two anchors, and a vibrating triple beam, which is clamped at both ends to the two proof masses. LPCVD silicon rich nitride is chosen as the resonant triple beam material, and parameter optimization of the triple-beam structure has been performed. The triple beam is excited and sensed electromagnetically by film electrodes located on the upper surface of the beam. Both simulation and experimental results show that the novel structure increases the scale factor of the resonant accelerometer, and ameliorates other performance issues such as cross axis sensitivity of insensitive input acceleration, etc.

## 1. Introduction

Resonant sensors have many advantages over the conventional type, such as high resolution, wide dynamic range, and quasi-digital nature of the output signal. One of the most commonly found silicon resonators is a microbeam clamped at both ends, which is sensitive to axial loads. This load can be made proportional to the physical parameters (acceleration, force, pressure etc.) via some kind of converting mechanism. To date, there have been several successful examples of resonant silicon accelerometers fabricated by both surface [1,2] and bulk micromachining technology [3–8]. A microleverage force amplifying mechanism is involved to realize an in-plane acceleration measurement through a double ended tuning fork (DETF) resonator and a cantilever-type accelerometer is used for out-of plane acceleration measurement. For the actuation of the resonator, the electrostatic [4, 6–8], piezoelectric, and electrothermal [3,5] methods are commonly used, and the sensing is typically accomplished by means of piezoresistive, capacitive,or piezoelectric methods. However, resonant silicon accelerometers utilizing microlever systems suffer from complexity of their fabrication processes and a low scale factor (less than 100 Hz/g), even if complicated multistage microleverage is employed. Cantilever-type accelerometers with thick proof masses and thin support beams usually have a larger scale factor (200 Hz/g [3]) but encounter cross-sensitivity problems, especially along the resonant beam direction due to the moment of the large proof mass.

**Figure 1.**
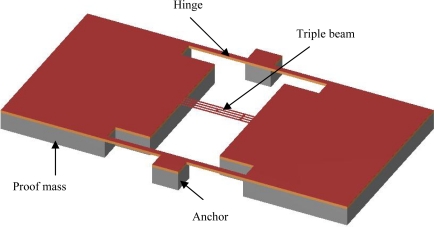
Schematic view of an electromagnetically excited resonant micro accelerometer.

In order to increase the scale factor and decrease cross-sensitivity, this paper presents a resonant microbeam accelerometer of a novel highly symmetric structure, shown schematically in Figure 1 and based on the same force amplifying mechanism as a cantilever-type accelerometer. The accelerometer consists of two identical proof masses, four supporting beams, two anchors and a vibrating triple beam, which is clamped at both ends to the two proof masses. LPCVD silicon rich nitride was chosen as the resonant triple beam material instead of heavily doped silicon [3,4] as it benefits from its high chemical resistance against KOH etching for beam release [9,10]. The triple beam is excited and sensed electromagnetically in a magnetic field by film electrodes located on the upper surface of the beam. When out-of plane acceleration is applied along the z direction, both proof masses move like in a cantilever type accelerometer, and either stretching or compression of the triple beam occurs. The mechanical load is converted into a change of resonant frequency of the beam. Arrangement of two symmetric proof masses helps to eliminate cross axis error. In order to predict its exact performance and to optimize the design, the commercial FEA software was used to analyze the resonant accelerometer. Numerical modeling was carried out on the resonant frequency shift due to applied acceleration. Simulation results show that the novel structure increases the scale factor of the resonant accelerometer, and effectively eliminates cross-sensitivity. Standard bulk silicon MEMS technology was used to manufacture the resonant accelerometer, which combines KOH anisotropic wet etching technology.

## 2. Design and Simulation

### 2.1 Chip design

The newly designed electromagnetically excited resonant micro accelerometer is shown in Figure 1. It includes two symmetric proof masses, connecting to two anchors via two pair of flexures to enable the proof mass to rotate about a hinge axis, and a triple-beam resonator clamped at both ends to the two proof masses.

**Figure 2.**
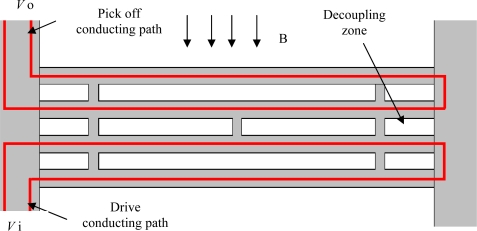
Schematic view of the triple-beam resonator.

**Figure 3.**
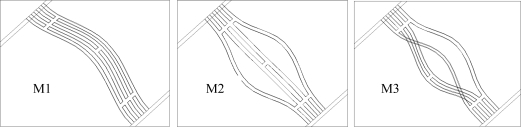
Three major flexure modes of triple-beam resonator.

To enhance the quality factor of the resonant micro accelerometer, a triple-beam resonator shown in Figure 2 has been designed. Actually, the triple beam consists of four identical beams with the central two beams coupled through a link, and they are joined together via a decoupling zone. The drive conducting path is located on the lower two beams, while the pick off conducting path is located on the upper two beams. An externally generated magnetic field B parallel to the chip plane is applied in a direction normal to the beam, and interacts with the AC drive current in the drive conducting path, causing the lower two beams to vibrate in opposite directions. The drive voltage is denoted as *V*i. Mechanical coupling forces the upper two beams to vibrate together, which causes a voltage (denoted as *V*o) to be generated across the pickoff circuit. This pickoff voltage is then sent to an amplifier by way of a positive feedback loop to form an oscillator. There are three major flexure modes for this triple-beam resonator which, which are shown in Figure 3. The fundamental flexure mode M1 with the lowest frequency in which all beams vibrate in phase is called common mode. The second mode denoted M2 is asymmetric, the outer beams oscillate in anti-phase and the central beam rests. The third mode M3 is called a differential one, which corresponds to the highest frequency. In the M3 state the vibrating direction of the two outer beams is opposite to the vibrating direction of the central beam, so that the shear force and moment coupling keep energy from leaking. It exhibits the highest Q factor and offers the highest resolution when used in sensors. The differential mode is desired and it can be tuned in closed-loop operation by a self-oscillating circuit.

Parameter optimization mainly concerns two factors as follows:

1) Resonant frequency distinction between M1and M3

If the resonant frequency of M1 is too close to the resonant frequency of M3, when the triple-beam is working in the resonant M3 state, energy dissipation would be significant and the quality factor would be relatively low. Based on this assertion, the resonant frequency of M1 should be set much lower than the resonant frequency of M3, which can be achieved by adjusting the length of the decoupling region.

2) Coupling effect

The coupling effect criterion is that the vibrating amplitude difference of the central beam and the outer beam of M3 should be set as a minimum in that when the central beam has twice the width of the two outer beams, if the central beam has the same vibrating amplitude as that of outer beams, the sheer force and moment applied by three beams on decoupling region can be cancelled, which means there will be no energy dissipation.

When the inertial force caused by the proof mass under out-of-plane (Z-direction) acceleration bends the leverage structure, the axial force, which is several times larger than the inertial force, because of the action of the leverage structure on the resonator, will modify its intrinsic resonant frequency.

The main advantage of this design is that unlike conventional cantilever-based micro accelerometers, the cross-sensitivity issue can be successfully resolved by means of only one resonator. Because of symmetric placement of two proof masses at both ends of the resonator, when this device is under in-plane (X-direction and Y-direction) acceleration, there is nearly no frequency shift though there is resonator deformation.

Another advantage of this structure lies in its triple-beam resonator excitation mechanism. The configuration of separate drive circuit and pick off circuit shown in Figure 2 ensures the triple-beam resonator is working in its desirable mode M3 by means of choosing the phase of exciting source, effectively disallowing the disturbing flexure modes M1 and M2. Further, the electrical cross-talk between input and output signals is also reduced by having separate drive and pick off circuits.

### 2.2 FEA Modeling

Finite element modeling is an integral and essential aspect of the design and development process for resonant beam sensors. Commercial FEA software has been used for the numerical modelling to examine this design. The central beam dimensions are 800 μm × 80 μm × 3 μm, those of the supporting beam 600 μm × 100 μm × 20 μm and the proof mass dimensions are 2 mm × 1.2 mm × 200 μm. The material properties for the modeling are given as: for silicon, density, 2.33×10^−15^ kg/μm^3^, Young’s Modulus, 1.65×10^5^ MPa, Poisson’s Coefficient, 0.22. for silicon nitride, density, 3.1×10^−15^ kg/μm^3^, Young’s Modulus, 3.85×10^5^ MPa, Poisson’s Coefficient, 0.245 [9].

**Figure 4.**
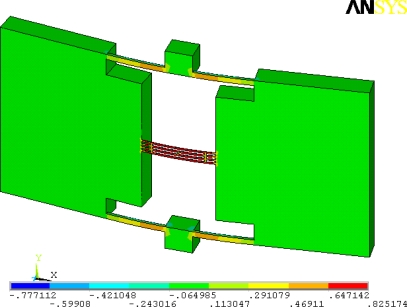
Distribution of *S*x under 1g acceleration load of Z direction for the novel structure.

First, computer simulations are carried out on axial stress distribution. Figure 4 depicts the stress contour along the direction of the beam length (in this case, *S*x) under an applied acceleration of 1g. The positive stress (tensile) is induced in the resonant beam.

Next, modal frequency analysis is carried out at different applied acceleration loads. The results are given in Table 1

**Figure 5.**
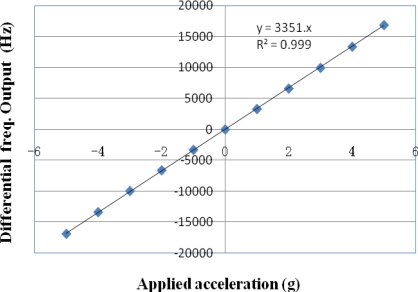
Simulated differential frequency output of a pair of identical sensors over the ±5g full scale.

Simulation results from this Table show that resonant frequencies M1–M3 change as applied loads vary, indicating vibrations of the two micro beams. Taking the shift of frequencies of each modal as output, one can get scale factors (denoted as *SF*) of each modal. *SF* (z) of M3 is about 1.6 kHz/g, *SF*(x) and *SF*(y) are less than 0.02 Hz/g. The cross axis sensitivities of X and Y axis are less than 15 ppm. To improve linearity and temperature stability of the accelerometer, a pair of identical sensors forms a differential frequency output. Since the measurement is based on the frequency difference, if there is any ambient temperature influence, the frequency drift induced on both beams will be the same. The frequency difference will be unchanged, guaranteeing a temperature independent acceleration sensing. Figure 5 is the simulated differential frequency output of M3 of two identical sensors due to applied acceleration over the full-scale operating range. As the applied acceleration changes from −5g to 5g, a scale factor of 3,350 Hz/g over the ±5g full scale is obtained.

## 3. Experiments and Results

**Figure 6.**
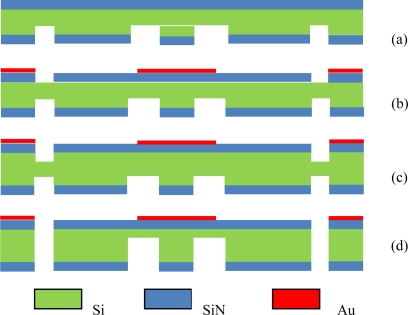
Fabrication process flow for a resonant accelerometer.

The starting materials are 3 inch p type <100> 300 μm silicon wafers, with the resistivity of 0.01 Ωcm. Standard bulk-silicon micromachining technology was used to manufacture this resonant micro accelerometer which combines ICP deep etching and KOH anisotropic wet etching technology. The basic fabrication steps are shown in Figure 6.

First, a thin silicon oxide (100 nm) and a thick low stress silicon rich SiN film (3 μm) are grown on both sides of the wafer by thermal oxidation and LPCVD methods, respectively. Using thick positive photoresist as the mask, SiN and SiO_2_ on the reverse side are removed by RIE etching selectively to define the proof mass, then, ICP deep etching is performed to etch silicon to the depth of 100 μm (a).

Second, by means of lift-off technology, gold film electrodes have been formed on the SiN film on the front side of the wafer (b). Then, using thick positive photoresist as the mask, SiN is removed by RIE etching selectively to define the SiN beam, supporting beam and the proof masses, and using the same mask, ICP deep etching is conducted to etch silicon to the depth of 40 μm (c) .

**Figure 7.**
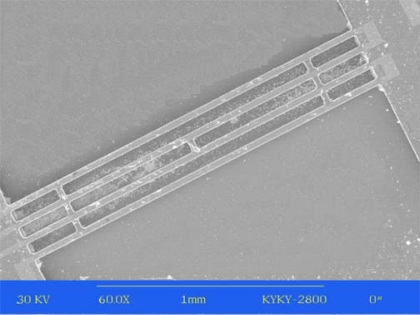
SEM of the triple beam.

Finally, the wafer is immersed in hot KOH solution, and the same etch is performed from the front and back sides of the wafer. The proof mass and suspension system are formed by time-controlled etching in KOH (d) and the SiN beams are released by undercutting. Figure 7 shows the SEM picture of the triple silicon nitride beam.

**Figure 8.**
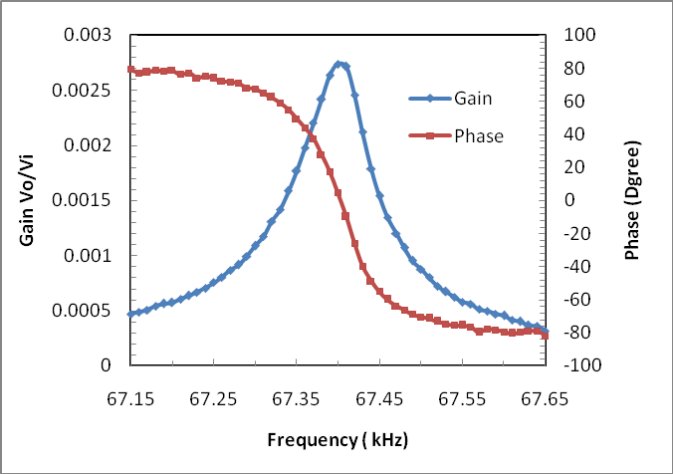
Measured gain and phase frequency response in air.

The frequency response function between *V*i and *V*o is measured with an HP3562A dynamic signal analyzer over the 67.15–67.65 kHz range in air, with *V*i =10 mV. Such voltage values result in a static power to the heater of about 5 mW. The results are shown in Figure 8, where a peak is visible corresponding to the natural frequency of the beam at 67.41 kHz. The value is in agreement with the prediction obtained by FEM, though slightly lower. This can be ascribed to inaccurate selection of the structure parameters of the materials, the microbeam boundary conditions that are more compliant than in the ideal clamped-clamped case, and to the effect of the temperature rise caused by the driven heater. It is estimated that the Q-factor of the resonator in air is 1,000, and can reach 10,000 in vacuum due to reduction of damping. The phase shift of nearly 180 degree indicates weak cross-talk between drive conducting path and pick off conducting path.

Static sensitivity measurements were performed in closed-loop operation by an off chip self-oscillating circuit. By turning the device in the gravitation field, several devices are measured, with sensitivities of one single chip ranging between 1,000 and 1,500 Hz/g, in agreement with the FEM predicated value, though slightly lower.

## 4. Conclusions

A silicon rich SiN resonant microbeam accelerometer of a novel highly symmetric structure is presented. The parameter optimization of the triple-beam resonator has been conducted in order to enhance the quality factor. The novel structure, which includes two symmetric proof masses at both ends of the resonator, can effectively eliminate cross-sensitivity. This excitation mechanism highly can avoid disturbing modes of the triple beam resonator, which further enhances the quality factor of the design. The major fabrication steps have been described in this paper and FEA simulation shows that the sensitivity of this design is more than 1,600 Hz/g over the ± 5g full scale. Preliminary measurements are in good agreement with simulated results. Further investigation into the acceleration sensing performance is being carried out.

## Figures and Tables

**Table 1.** Simulation results of modal frequency analysis at different applied acceleration loads.

**Loads**	**0g**	**1g (Z)**	**100g (X)**	**100g (Y)**

M1	40773	42385	40774	40775
M2	65798	67250	65800	65799
M3	73722	75336	73722	73722
